# Recent Advances in Biomarkers and Regenerative Medicine for Diabetic Neuropathy

**DOI:** 10.3390/ijms22052301

**Published:** 2021-02-25

**Authors:** Yoshikai Fujita, Tatsufumi Murakami, Akihiro Nakamura

**Affiliations:** 1 Division of Pharmaceutics, Department of Pharmacology, Toxicology and Therapeutics, School of Pharmacy, Showa University, 1-5-8 Hatanodai, Shinagawa-ku, Tokyo 142-8555, Japan; hironak@pharm.showa-u.ac.jp; 2 Department of Neurology, Kawasaki Medical School, 577 Matsushima, Kurashiki, Okayama 701-0192, Japan; tatsum@med.kawasaki-m.ac.jp

**Keywords:** diabetic neuropathy, biomarker, regenerative medicine, supplementation therapy with exogenous, cytokines cell transplantation therapy, exosome

## Abstract

Diabetic neuropathy is one of the most common complications of diabetes. This complication is peripheral neuropathy with predominant sensory impairment, and its symptoms begin with hyperesthesia and pain and gradually become hypoesthesia with the loss of nerve fibers. In some cases, lower limb amputation occurs when hypoalgesia makes it impossible to be aware of trauma or mechanical stimuli. On the other hand, up to 50% of these complications are asymptomatic and tend to delay early detection. Therefore, sensitive and reliable biomarkers for diabetic neuropathy are needed for an early diagnosis of this condition. This review focuses on systemic biomarkers that may be useful at this time. It also describes research on the relationship between target gene polymorphisms and pathological conditions. Finally, we also introduce current information on regenerative therapy, which is expected to be a therapeutic approach when the pathological condition has progressed and nerve degeneration has been completed.

## 1. Introduction

Diabetic neuropathy is the earliest and most frequent complication of the three major complications associated with diabetes, but it is often not diagnosed until the disease has seriously progressed [1]. Therefore, early diagnosis is extremely important for care and treatment. This complication is peripheral neuropathy with predominant sensory impairment, and its symptoms are hyperesthesia, pain, and a gradual loss of sensation due to the loss of nerve fibers. When hypoalgesia occurs, trauma and mechanical irritation cannot be noticed; as a result, foot ulcers and gangrene may occur, leading to amputation of the lower limbs [2]. Therefore, since this disease imposes a physical and mental burden on patients and their families and decreases the quality of life, it is important to develop diagnostic methods with high sensitivity for its early detection. It has been clarified that the onset and progression of neuropathy can be suppressed by strictly controlling the blood glucose levels of diabetic patients over long periods of time [3,4]; however, it is difficult to completely prevent neuropathy. Currently, symptomatic treatment is the only option, as there is no reliable and effective treatment method.

It has been reported that up to 50% of diabetic peripheral neuropathy cases can be asymptomatic [5]. Therefore, together with clinical symptoms and neurological findings for the early detection of diabetic neuropathy, more sensitive and convenient biomarkers that detect the severity or stage progression are required. Information on the pathogenic mechanism is required for the development of biomarkers; however, there are different theories on the pathogenic mechanism of diabetic neuropathy, which makes the study of its mechanism very complicated [6]. Due to glucose uptake independent of insulin, metabolic changes occur in peripheral nerve tissue when hyperglycemic conditions persist. Due to damage to vascular endothelial cells of neurotrophic blood vessels and degeneration of nerve fibers (axons and Schwann cells), diabetic neuropathy finally appears. (1) Promotion of polyol metabolism, (2) promotion of the production of advanced glycation end products (AGEs), (3) an increase in free radicals, (4) a decrease in NO levels, and (5) promotion of the activity of protein kinase Cβ (PKCβ) have been proposed to be involved in metabolic changes. It is reported that they function synergistically to cause this disease. Since biomarkers reflect the complexity of these etiologies and the progression of the pathological condition, several types of factors have been identified as potential biomarkers of diabetic neuropathy. These include factors closely linked to the etiology, those associated with the progression of pathological conditions such as inflammation, and molecules involved in the cellular response to vascular endothelial cell damage and neurodegeneration. 

In addition to biomarkers localized in intracellular organelles or specific cells, this review focuses on systemic biomarkers, such as serum, which are less invasive and can be easily quantified. Other excellent reviews [7,8,9] also describe local biomarkers. In addition, since biomarkers are molecules whose levels fluctuate during disease progression, they are often the cause on their own; hence, supplementing them can slow down or restore the progress of disease. This review also outlines current information on regenerative therapy, which is expected to be a treatment method when the condition progresses and nerve degeneration is complete.

## 2. Biomarkers for Diabetic Neuropathy

In this review, biomarkers for diabetic neuropathy are divided into four groups: (a) AGE-related molecules (methyl glyoxal and glyoxalase I), (b) molecules that participate in the progression of inflammation (Toll-like receptors, TNF-α, miR-146a, adiponectin, etc.), (c) molecules associated with nerve damage (nerve specificity enolase and semaphorin), and (d) molecules involved in nerve protection (nerve growth factor and HSP27). Group (a) is considered the causative agent of the onset of diabetic neuropathy, and the biomarkers from groups (b)–(d) are regarded as those that manifest in the late stage of disease progression. Table 1 summarizes the biomarkers for diabetic neuropathy covered in this section.

### 2.1. AGE-Related Molecules

#### 2.1.1. AGEs and Their Precursors

**Figure 1:**
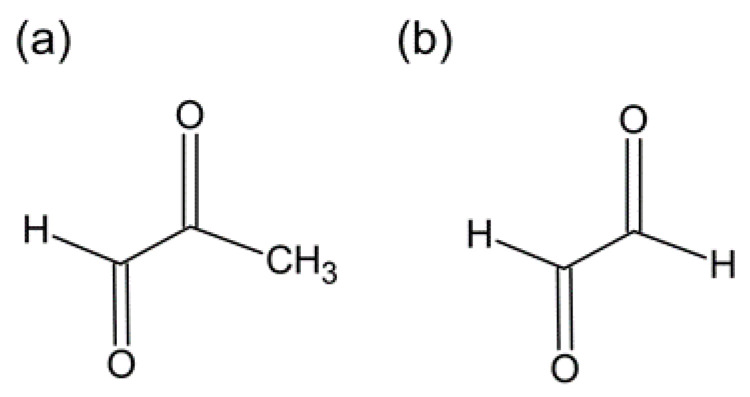
Advanced glycation end products (AGE)-related molecules. (**a**) Methylglyoxal, (**b**) α-oxoaldehyde.

Non-enzymatic glycation of proteins has been established as a major cause of diabetic complications, such as nephropathy and macroangiopathy. Excessive accumulation of AGEs is also observed in peripheral nerve tissue in diabetic neuropathy, which has been shown to correlate with a decrease in the number of nerve fibers [10]. Impaired axonal transport due to the modification of major axonal cytoskeletal proteins, such as tubulin, by AGEs [11] and impaired axon regeneration due to modification of the basement membrane protein laminin [12] are thought to contribute to the development of neural lesions. In addition, in knockout mice lacking AGE receptors (AGEs: RAGE), the loss of pain perception was prevented, demonstrating that RAGE expression is directly involved in the onset of neuropathy [13]. Accumulation of AGEs in nerve tissue is considered to be a major cause of the onset and progression of neuropathy in humans. However, the measurement of AGEs in tissues is difficult in terms of the collection and quantification of samples and does not seem to be suitable as a biomarker. On the other hand, reactive dicarbonyls, such as methylglyoxal and α-oxoaldehyde (Figure 1), which are precursors of AGEs, are receiving much attention as biomarkers for predicting the onset and progression of diabetic neuropathy [14]. Bierhaus et al. [15] reported that methylglyoxal depolarizes sensory nerves, causes post-translational modification of the voltage-gated sodium channel Nav 1.8, and induces hyperalgesia. Furthermore, it was reported that the cold receptor channel transient receptor potential cation channel A1 (TRPA1) was also activated, causing temperature and mechanical hyperalgesia [16]. When methylglyoxal was administered to mice, a decrease in nerve conduction velocity and promotion of the secretion of calcitonin gene-related peptides from cutaneous nerve endings were observed, and hyperalgesia to heat stimulation and mechanical stimulation was induced. Similar changes were observed in streptozotocin-induced and hereditary diabetic mouse models but not in Nav 1.8 knockout mice. Likewise, microinjection of methylglyoxal into the skin of healthy human volunteers also induces chemical pain sensations and thermal hyperalgesia [17]. These findings strongly suggest that the mechanism of hyperalgesia in diabetic neuropathy is due to methylglyoxal itself. Furthermore, recent reports from animal experiments have shown that both hyperalgesia and itching and hypoalgesia are induced by direct intradermal or intrathecal injection [18]. Therefore, methylglyoxal can reproduce various symptoms of diabetic neuropathy that resemble clinical symptoms. Hansen et al. reported no association between serum methylglyoxal and the occurrence of diabetic peripheral neuropathy in a cohort of well-treated patients with short-term type 2 diabetes [19]. On the other hand, Andersen et al. [20] reported a significant association, suggesting that higher levels of methylglyoxal are identified as risk factors for the development of diabetic neuropathy Thus, there are discrepancies in the results depending on the length of the survey period, and a detailed examination is awaited.

#### 2.1.2. Glyoxalase I (GLO I)

It is well known that there is a pathway for degrading reactive dicarbonyls such as methylglyoxal in vivo by glyoxalase I (GLO I), the rate-determining enzyme [21]. Recently, it has been shown that the expression of GLO I varies in sensory neurons from two inbred strains of mice. Neuropathic symptoms, such as a decreased pain threshold and decreased intraepidermal nerve fiber density, observed in diabetic conditions are significantly suppressed in mice with high GLO I activity [22]. From these results, it can be assumed that the presence or absence of GLO I gene polymorphism affects the onset of diabetic neuropathy in humans. Multiple GLO I gene polymorphisms are known to exist, some of which have decreased enzymatic activity, suggesting that there are individual differences in GLO I activity [23]. A recent report showed a significant correlation between decreased GLO I activity in serum samples and painful diabetic neuropathy [24]. In addition, Groener et al. reported that the incidence of diabetic neuropathy was predominantly higher in type 2 diabetic patients with a mutant homozygous for C332C in the GLO I gene [25]. Thus, GLO I activity may be a useful biomarker. 

### 2.2. Molecules That Participate in the Progression of Inflammation 

#### 2.2.1. Toll-Like Receptor (TLR)

Toll-like receptors (TLRs) are receptors that play an important role in the innate immune response [26]; among these, TLR4 is associated with many diseases of the immune system [27]. On the other hand, in the peripheral nerves of diabetic humans and animals, infiltration of inflammatory cells, such as macrophages and lymphocytes, is observed, and the production of cytokines, such as tumor necrosis factor (TNF-α) and interleukin (IL), is enhanced [28]. TLR2/4-knockout mice are less likely to develop neuropathy due to the ingestion of a high-fat diet, suggesting a relationship with the pathogenic mechanism of diabetic neuropathy [29].

Zhu et al. investigated the expression levels of TLR4 and its downstream genes in human peripheral blood mononuclear cells collected from patients with type 2 diabetes [30]. It was observed that the expression levels of TLR4 were increased in diabetic patients who developed neuropathy compared to those in healthy subjects and diabetic patients who did not develop neuropathy. Furthermore, the levels of TNF-α and IL-6 in serum were also significantly increased in diabetic patients who developed neuropathy. These results suggest that TLR4 may be a useful marker for diabetic neuropathy. However, this study was conducted only on patients with type 2 diabetes, and the number of subjects was relatively limited; therefore, further research is needed. Another group has also reported elevated levels of TNF-α in the serum samples of patients with diabetic neuropathy [31]. 

There are multiple gene polymorphisms in TLR4; the TLR4 Asp299Gly gene polymorphism participates in the decrease in IL-8 production after LPS stimulation [32]. In patients with type 2 diabetes with this polymorphism, the incidence of diabetic neuropathy was reduced [33]. However, no consensus has been reached because such a correlation was not found in reports from other groups [34]. 

#### 2.2.2. Adiponectin

Adiponectin is an adipocytokine secreted by adipocytes and is a 30 kDa protein. Adiponectin plays various roles in human metabolism, such as lipid regulation, glucose metabolism, and increased insulin sensitivity [35]. Several studies have investigated the relationship between serum adiponectin levels and diabetic nerve injury. Although there is an association between them, corroborating results have not always been obtained. For example, a cross-sectional study in India examined serum adiponectin in 487 patients with type 2 diabetes and found that diabetic patients with neuropathy had significantly higher levels of adiponectin than those without it [36]. Similarly, a study by Pradeepa et al. reported that a high level of adiponectin was associated with an increased incidence of neuropathy [37]. On the other hand, although serum adiponectin levels are associated with diabetic neuropathy, the KORA F4/FF4 study reported that decreased, rather than elevated, serum adiponectin levels were associated with diabetic peripheral neuropathy incidence [38]. Therefore, these conflicting studies suggest that different genetic backgrounds and target ages due to diversity in the ethnic composition of participants affect adiponectin levels in serum. However, these results also suggest that there is a relationship between serum adiponectin levels and the development of diabetic neuropathy. Therefore, adiponectin remains one of the promising biomarkers for the future, and it is expected that more standardized research will be conducted across different racial groups. 

#### 2.2.3. MicroRNAs (miRNAs)

miRNAs are non-coding single-stranded RNA, about 20–22 bases in length, which control gene expression by suppressing degradation or translation by complementarily binding to the 3’ untranslated region of the target mRNA [39]. miRNAs are attracting attention as new biomarkers, as their expression patterns are relevant to pathophysiological processes.

miR-146a is one of the miRNAs whose expression levels are reduced in the serum samples of diabetic patients [40]. Wang et al. reported that hyperglycemia downregulated miR-146a expression and elevated interleukin-1 receptor activated kinase (IRAK1) and tumor necrosis factor receptor-associated factor 6 (TRAF6) levels in dorsal root ganglia (DRG) neurons [41]. Furthermore, miR-146a has been shown to play an important role in mediating DRG neuron apoptosis under hyperglycemic conditions.

Other candidate miRNAs that could be useful biomarkers are being explored. Massaro et al. recently performed the miRNA expression profiling of peripheral blood mononuclear cells collected from 63 diabetic patients, classified them according to the type of complication [42], and identified the miRNA groups that were specifically upregulated in the diabetic neuropathy group (miR-125a-5p, miR-145-3p, miR-99b-5p, and miR-873-5p). Furthermore, serum miR-518d-3p and miR-618 were upregulated in patients with diabetic peripheral neuropathy compared to individuals without microvascular complications in a cohort study of patients with type 1 diabetes [43]. These data suggest that circulating miRNAs may serve as potential biomarkers for the diagnosis of diabetic neuropathy in the future.

### 2.3. Molecules Associated with Nerve Damage

#### 2.3.1. Neuron-Specific Enolase (NSE)

Nerve-specific enolase (NSE) is a glycolytic enzyme that exists specifically in nerve tissue and shows a high positivity rate, especially in small-cell lung cancer and neuroblastoma. Therefore, NSE is widely used as a tumor marker for detecting these diseases [44,45,46]. Li et al. investigated the relationship between blood NSE levels and diabetic neuropathy because the synthesis of these enzymes may be altered during the process of degeneration and regeneration of peripheral nerves due to the oxidative stress caused by chronic hyperglycemia [47]. The serum NSE levels were slightly higher in the type 1 and type 2 diabetic groups than in the control group. In particular, they increased in the group with neuropathy. This relationship was independent of fasting blood glucose, HbA1c, duration of illness, diabetic type, age, gender, renal function, and serum NSE levels, which were associated with the degree of neuropathy. In addition, it has recently been reported that the value decreased not only with the onset and progression but also with the improvement in neuropathy in response to treatment [48]. This result suggests that NSE may be a marker for predicting therapeutic effects as well as for early detection of diabetic neuropathy; however, future studies, including large-scale clinical trials, are awaited.

#### 2.3.2. Semaphorins

Semaphorins are a large family of proteins originally identified as axon guidance factors of the developing nervous system. They are multifunctional proteins that play important roles in various biological processes, such as immune responses, organogenesis, and angiogenesis [49,50,51]. Since these proteins are also involved in axon guidance during the regeneration process after damage to peripheral nerves, they may be ideal candidates as biomarkers for diabetic neuropathy. Several studies reported that these proteins were induced in peripheral nerves distal to a transection or crush injury in a ligation rat model [52,53]. In addition, the administration of recombinant Semaphorin 3A (Sema3A) protein attenuated mechanical allodynia and heat hyperalgesia in chronic constriction injury (CCI) rats [54]. In line with these observations, Wu et al. reported that higher Semaphorin 3A expression was accompanied by reduced intraepidermal nerve fiber density in the skin of diabetic patients compared with that in control subjects [55]. 

However, there is no information about changes in the expression of these genes in diabetic neuropathy animal models. Therefore, we investigated the expression levels of six types of semaphorin molecules in diabetic neuropathy model mice. As the results indicate, no statistically significant changes were observed in the expression levels of any of the molecular species (unpublished data).

### 2.4. Molecules Involved in Nerve Protection

#### 2.4.1. Nerve Growth Factor (NGF)

Neurotrophic factors are growth factors that can promote the survival and growth of neurons. In diabetic mice, decreased expression of neurotrophic factors, such as nerve growth factor (NGF), in nerve tissue or surrounding tissues was observed at the onset of neuropathy. Furthermore, the administration of exogenous nerve growth factor leads to an improvement in diabetic neuropathy [56]. Therefore, neurotrophic factors are considered to be candidates for biomarkers for diabetic neuropathy, but there are not many reports of such studies in humans. To date, there have been three reports of significantly reduced blood NGF levels in diabetic patients with neuropathy [57,58,59]. However, serum NGF levels were not associated with neuropathy severity in patients with diabetic peripheral neuropathy in some studies [59]. Therefore, it remains unknown whether NGF is useful enough for predicting diabetic neuropathy and understanding its pathophysiology. Further studies are required to clarify these points.

#### 2.4.2. Heat Shock Protein (HSP27)

HSP27 is a small heat shock protein that plays an important role in cell protection under stress. Increased levels of HSP27 have been observed in DRG cells of the spinal cord in diabetic mice [60]. In addition, this protein plays a role in protecting Schwann cells from apoptosis [61]. Although several studies have investigated the relationship between serum HSP27 levels and patients with diabetic neuropathy [62,63,64], these results are conflicting. One study reported a correlation between blood HSP27 levels in patients and the occurrence of diabetic polyneuropathy. However, other reports for HSP27 were not in agreement. Further investigation, such as prospective cohort studies, is necessary to clarify this.

## 3. Current Status of Regenerative Medicine

### 3.1. Supplementation Therapy with Exogenous Cytokines

Since there is no effective therapy for diabetic neuropathic pain, symptomatic treatment is the only option. In the case of diabetic neuropathy progress in which vascular and nerve degeneration is complete, impaired nerve function cannot be restored by these treatments. Regenerative medicine is an alternative method that has the potential to restore nerve damage caused by diabetic neuropathy. It can be divided into two approaches: (1) supplementation therapy with exogenous cytokines and (2) cell transplantation therapy. In the former, several cytokines have been investigated for the treatment of diabetic neuropathy. These proteins include vascular endothelial growth factor (VEGF) [65], hepatocyte growth factor (HGF) [66], basic fibroblast growth factor (bFGF) [67], and NGF [56]. It has been shown that the gene transfer of these factors or administration of recombinant proteins elicits several limited therapeutic effects. Although most studies are limited to animal experiments, improvements in nerve blood flow and nerve function through angiogenesis in perineural tissues have been observed. For VEGF, a phase II clinical trial was conducted in humans using an expression plasmid [68]. Although no significant difference was observed in the improvement of sensory function and nerve conduction velocity 6 months after intramuscular injection, the subjective symptom score improved in that study. The intramuscular administration of an engineered zinc finger protein activator of endogenous VEGF-A showed improvement in the nerve function of the treated mice [69]. However, there are several problems with this therapy. Ectopic angioplasty cannot be fully avoided using VEGF gene therapy. VEGF expression is associated with the onset of diabetic retinopathy [70], and administration of VEGF may exacerbate retinopathy. 

We have confirmed the therapeutic effect of VEGF and its homolog placental growth factor 2 (PlGF-2) gene transfer by electroporation in diabetic mice [71,72]. We also found that some isoforms (VEGF-121, PLGF-1) with angiogenic activity do not have the effect of improving nerve function. This result suggests that vascular growth factor may improve nerve function by a pathway different from the VEGF pathway, which is not relevant to angiogenesis. Therefore, if this putative mechanism can be elucidated, it will help in the development of therapeutic agents for new strategies.

### 3.2. Cell Transplantation Therapy

Several cell lines have been reported as a source for cell transplantation therapy. These cell lines include mononuclear cells [73], mesenchymal stem cells [74], endothelial progenitor cells [75], etc. In these studies, restoration of nerve function was attributed to several mechanisms induced by cell transplantation. It has been suggested that these cells produce angiogenesis and nerve growth factors at the transplant site and may directly affect tissue repair. 

Supplementation therapy with cytokines has the problem of low expression efficiency of the introduced gene vector, and the expression of the gene is transient. On the other hand, cell transplantation therapy, a system that continuously expresses the factors necessary for tissue regeneration, is very attractive, but there is concern about its safety because the long-term continuous expression of biologically active substances may cause unexpected side effects. It is important that data on clinical efficacy and safety be accumulated for both therapies. 

Exosomes are vesicles secreted by cells with a size of approximately 30–120 nm and have a lipid bilayer structure [76]. When first discovered, exosomes were thought to be garbage bags for discarding unnecessary cellular components. They include not only lipids and proteins but also nucleic acids, such as miRNA and mRNA. Recent studies have revealed that exosomes play an important role as signal mediators for cell–cell communication. In addition, they have regenerative properties, which encourage their application for therapeutic purposes. This approach, using exosomes, is considered to have the advantages of both cytokine replacement and cell therapy. 

The application of exosomes to therapy for diabetic neuropathy has recently been reported. Lopez-Verrilli et al. observed that Schwann cell-derived exosomes (SC-exos) were internalized by DRG axons and promoted axon regeneration [77]. Wang et al. reported that SC-exos have the effect of increasing the density of intraepidermal nerve fibers in the footpad and reduce myelin damage in the sciatic nerve, which are neuropathy symptoms found in diabetic neuropathy mice [78]. On the other hand, SC-exos cultured under high-glucose conditions tended to reduce epidermal nerve fibers and promote the development of diabetic neuropathy. SC-exos contained miR-21, miR-27a, and miR-146a, but the miR-27a-depleted exosomes lost their effect of promoting axonal growth and Schwann cell migration in DRG neurons. Therefore, it is believed that miR-27a plays an important role in the improvement of nerve function. 

Like SC-exos, exosomes derived from mesenchymal stromal cells (MSCs) have also been found to have a significant improvement effect on neuropathy symptoms after their administration in mice [79]. This effect is believed to be due to inhibition of the inflammatory response by MSC-exosomes and neurovascular remodeling.

As described above, the effectiveness of regenerative therapy for diabetic neuropathy has been proven in animal studies as summarized in Table 2. However, several problems need to be solved before medical application. In cytokine replacement therapy, a precise gene delivery system is necessary for gene therapy, which sustains the effect of cytokines at the target region. In addition, the efficacy of gene transfer is expected to have a limited duration of action when compared with cell transplantation therapy. 

Cell transplantation therapy presents several problems in clinical application, such as tumorigenesis, immunological rejection, and ethical issues. Moreover, it is important to ensure the safety and quality of cells that will be therapeutically effective. Compared to the above two methods, therapy using exosomes seems to be more advantageous because it contains microRNA, cytokines, and other biologically active molecules, which are necessary substances for cell therapy itself. However, this approach also has several challenges, such as establishing cell lines to produce enough exosomes and standardizing the quality of exosomes.

Despite these challenges, the fact remains that regenerative therapy is a promising treatment for patients with advanced disease stages. Thus, it is expected that data on clinical efficacy and safety will be accumulated for each method.

## 4. Conclusions

Diabetic neuropathy reduces the quality of life of patients. Therefore, the development of diagnostic methods for the early diagnosis of diabetic neuropathy is required. However, as described in this review, although there have been multiple candidates for biomarkers, none of them can be applied in full in clinical practice. One of the causes seems to be that the etiology of this disease is multifactorial, and in addition, the pathological condition differs depending on the course of a disease from the onset, making it difficult to approach clinically. Therefore, in order to establish a biomarker for diabetic neuropathy, it is important to select the pathological condition according to appropriate diagnostic criteria and examine it using the same method for each stage. Fortunately, the progress of comprehensive analysis technology, represented by microarray analysis and mass spectrometry in recent years, is remarkable, and we hope that new biomarkers will be discovered by utilizing these methods.

## Figures and Tables

**Table 1:** Biomarkers for diabetic neuropathy mentioned in this review.

Biomarker Candidate	Sample Source	Quantitative Method	Function	Changes during Neuropathy	Literature
**(a) AGEs related**					
Methylglyoxal	Human serum	HPLC	Post-translational modification of voltage-gated sodium channel Nav 1.8 causes hyperalgesia. In addition, it activates TRPA1 and induces temperature and mechanical hyperalgesia.	When administered to mice, hyperalgesia due to thermal and mechanical stimulation is induced.	[14,15,16,17,18,19,20]
Glyoxalase I (GLO I)	Mouse DRG Human serum	Colorimetric method	A rate-limiting enzyme that decomposes reactive dicarbonyls such as methylglyoxal	The neuropathic symptoms observed in diabetic conditions are significantly suppressed in mice with high GLO I activity. In humans, decreased GLO I activity is also significantly correlated with the frequency of painful neuropathy	[21,22,23,24,25]
**(b) Inflammation-related molecules**					
TNF-α	Human serum	ELISA	Expression of cell adhesion molecules and induction of apoptosis Increased inflammatory mediator	It is elevated in type 2 diabetic patients in the control group and in the diabetic and neuropathy group compared to the diabetic group	[28,29]
TLR4	Human peripheral blood mononuclear cells	qPCR	Receptors involved in innate immunity	It is elevated in type 2 diabetic patients in the control group and in the diabetic and neuropathy group compared to the diabetic group	[30,31,32,33,34]
Adiponectin	Human serum	ELISA	Adipocytokines produced and secreted by adipocytes	There is a significant difference between the control group and the diabetic group with neuropathy compared with the control group in type 2 diabetic patients (the distinction differs depending on the report).	[36,37,38]
miR-146a	Human Serum Mouse DRG	qPCR	Negative feedback on inflammatory response	It is elevated in patients with type 2 diabetes, but its association with neuropathy has not been investigated. In diabetic neuropathy mice, the expression level in the DRG is reduced, and the forced expression in the DRG after culture acquires resistance to hyperglycemia-induced apoptosis.	[40,41]
**(c)Molecules associated with nerve damage**					
Nerve-specificenolase (NSE)	Human serum	ELISA	Glycolytic enzymes specific to nerve tissue (Leakage due to nerve damage)	It is elevated in type 1 and type 2 diabetic patients compared to the control group, especially in the group with neuropathy.	[47,48]
Semaphorin	Mouse sciatic nerve	qPCR	Nerve axon elongation guidance factor	These mRNAs are induced in the sciatic nerve in a rat sciatic nerve ligation model.	[52,53,54,55]
**(d) Molecules involved in neuroprotection**					
NGF	Human serum	ELISA	Neurotrophic factors involved in nerve regeneration	It is lower in type 2 diabetic patients in the control group and in the diabetic and neuropathy group than in the diabetic group	[56,57,58,59]
HSP27	Human serum	ELISA	Cell protective factors in the presence of stress	It is elevated in type 1 and type 2 diabetic patients compared to the control group, especially in the group with neuropathy.	[60,61,62,63,64]

**Table 2:** Regenerative medicine mentioned in this review

Supplementation Therapy with Exogenous Cytokines			
	Method	Clinical Significance	Literature
hepatocyte growth factor (HGF)	nonviral liposome-mediated gene transfer	Improvement in nerve conduction velocity	[66]
basic fibroblast growth facto (bFGF)	intramuscular injection of recombinant bFGF protein	Improvement in the motor nerve conduction velocity of the sciatic nerve and in sciatic nerve blood flow	[67]
nerve growth factor (NGF)	recombinant protein	Improvement of thermal allodynia in streptozotocin-induced diabetic rats.	[56]
placental growth factor 2 (PLGF-2)	intramuscular gene transfer of plasmid DNA by electroporation	Improved hypoalgesia in diabetic mice Restoration sensory nerve function	[72]
**Cell transplantation therapy**			
	cell source	Clinical Significance	Literature
mononuclear cells	bone marrow	Improvement of mechanical hyperalgesia and cold allodynia in streptozotocin-induced diabetic rats. Improvement in sciatic motor nerve conduction velocity, sensory nerve conduction velocity	[73]
mesenchymal stem cells	bone marrow	Improvement of mechanical hyperalgesia in streptozotocin-induced diabetic rats. Restoration of nerve conduction velocity and sciatic nerve blood flow	[74]
endothelial progenitor cells	umbilical cord blood	Improvement in sciatic motor nerve conduction velocity and sciatic nerve blood flow	[75]
**Exosome**			
	exosome source	Clinical Significance	Literature
Schwann cell-derived exosomes	Schwann cell	Improvement in sciatic nerve conduction velocity and increasing thermal and mechanical sensitivity in diabetic mouse	[78]
Mesenchymal stromal cell-derived exosomes	bone marrow	Improvement of thermal and mechanical sensitivity in diabetic mouses. Improvement in sciatic motor nerve conduction velocity, sensory nerve conduction velocity	[79]

## Data Availability

The data presented in this study are available on request from the corresponding author.
